# Restoring Septohippocampal Cholinergic Signaling Rescues Surgery‐Induced Neurogenesis and Memory Deficits in Aged Mice

**DOI:** 10.1111/acel.70574

**Published:** 2026-06-04

**Authors:** Lei Lei, Qingsheng Meng, Xiaoyu Hu, Jinjin Yang, Ni Du, Songxue Su, Kenji Hashimoto, Jing Cao, Jian‐jun Yang

**Affiliations:** ^1^ Department of Anesthesiology, Pain and Perioperative Medicine The First Affiliated Hospital of Zhengzhou University Zhengzhou China; ^2^ Henan Province International Joint Laboratory of Pain, Cognition, and Emotion Zhengzhou China; ^3^ Department of Anesthesiology and Perioperative Medicine, Henan Provincial People's Hospital People's Hospital of Zhengzhou University Zhengzhou China; ^4^ School of Basic Medical Sciences Zhengzhou University Zhengzhou China; ^5^ Center for Forensic Mental Health Chiba University Chiba Japan

**Keywords:** adult hippocampal neurogenesis, cholinergic signaling, postoperative cognitive dysfunction, septohippocampal pathway

## Abstract

Central cholinergic dysfunction contributes to postoperative cognitive dysfunction (POCD) in the aged, yet the specific circuit mechanisms remain poorly defined. Here, we investigated the role of cholinergic projections from the medial septum/vertical limb of the diagonal band (MS/vDB) to the dentate gyrus (DG) in surgery‐induced cognitive and neurogenic impairments in aged mice. Using a laparotomy model, we found that surgery led to memory deficits and suppressed adult hippocampal neurogenesis (AHN). In vivo fiber photometry revealed reduced hippocampal acetylcholine (ACh) release and decreased activity of MS/vDB cholinergic neurons. Local potentiation of DG cholinergic signaling with the acetylcholinesterase inhibitor galantamine rescued both memory deficits and AHN. Whereas acute chemogenetic activation of MS/vDB → DG projections alleviated anxiety‐like behavior, only sustained activation of this pathway restored memory function and promoted neurogenesis. Together, these results establish a causal link between loss of MS/vDB → DG cholinergic signaling and POCD‐related phenotypes, and demonstrate that restoring this specific input rescues neurogenesis and memory, highlighting its therapeutic potential.

## Graphical abstract

Surgery‐induced MS/vDB → DG cholinergic decline impairs hippocampal neurogenesis and memory in aged mice; galantamine‐mediated cholinergic potentiation or sustained chemogenetic activation rescues these deficits, highlighting therapeutic potential for POCD.

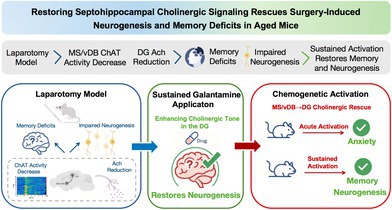

## 1 Introduction

Postoperative cognitive dysfunction (POCD) is a prevalent and serious complication adversely affecting the quality of life and long‐term survival of elderly patients (O'Brien et al. 2017). A key pathological feature of POCD is cholinergic system dysfunction (Xu et al. 2019). Early clinical observations indicated that preoperative administration of cholinergic antagonists increases the risk of postoperative cognitive impairment (Moller et al. 1998; Evered and Silbert 2018). Consistently, postmortem studies in POCD patients and animal models have revealed significant reductions in crucial synaptic cholinergic markers, including choline acetyltransferase (ChAT), acetylcholine (ACh), and the vesicular acetylcholine transporter (VAChT) (Chen et al. 2020; Lin et al. 2020). Although acetylcholinesterase inhibitors (e.g., galantamine and donepezil) have shown modest efficacy in mitigating POCD (Doraiswamy et al. 2007; Wang et al. 2019), their therapeutic benefits remain limited, suggesting that merely amplifying global cholinergic neurotransmission may be insufficient. Consequently, a deeper understanding of the mechanisms underlying cholinergic circuit dysfunction in POCD is urgently needed, particularly regarding how anesthesia and surgery impact distinct cholinergic projections and how these alterations contribute to specific behavioral deficits.

The medial septum and vertical limb of the diagonal band (MS/vDB) comprise the primary source of cholinergic projection neurons in the basal forebrain, and their integrity is strongly correlated with cognitive performance (Ananth et al. 2023). Despite this, the precise role of MS/vDB cholinergic neurons in POCD pathogenesis remains unclear. Ascending projections from the MS/vDB provide the major cholinergic input to the hippocampus via the septohippocampal pathway (Shivakumar et al. 2024; Király et al. 2023). While the hippocampus is widely recognized as a critical region in POCD, functional changes within the MS/vDB‐hippocampal cholinergic circuit following anesthesia and surgery have been scarcely investigated. Furthermore, the dentate gyrus (DG), and specifically its subgranular zone (SGZ), is a primary niche for adult hippocampal neurogenesis (AHN) (Zanirati et al. 2023). Although impaired AHN has been implicated in POCD (Kong et al. 2024), the specific mechanisms disrupting the neurogenic microenvironment are not well defined. Given emerging evidence that cholinergic signaling promotes neural progenitor proliferation and survival (Madrid et al. 2021; Chen et al. 2024), we hypothesized that surgery‐induced suppression of neurogenesis may be directly linked to the impairment of septohippocampal cholinergic input and dysregulation of acetylcholine receptors.

This study investigated the role of the MS/vDB‐hippocampal cholinergic circuit in postoperative cognitive decline and impaired neurogenesis in aged mice. We established a POCD model via exploratory laparotomy and employed a multi‐faceted approach to address the existing knowledge gaps. First, using in vivo fiber photometry, we dynamically monitored the activity of MS/vDB cholinergic neurons and ACh release in the DG during cognitive tasks following surgery. Second, we pharmacologically elevated ACh levels in the DG using galantamine to assess its impact on cognitive deficits and AHN. Finally, we used chemogenetic tools to selectively activate MS/vDB → DG cholinergic projections and locally applied the nicotinic acetylcholine receptor (nAChR) antagonist mecamylamine (MEC) to delineate the underlying receptor mechanisms. Our findings provide compelling evidence for a causal relationship between the loss of MS/vDB → DG cholinergic signaling and postoperative cognitive and neurogenic deficits, while also elucidating the therapeutic potential of restoring this specific pathway.

## 2 Methods

### 2.1 Animals

All animal care and experimental procedures were approved by the Animal Care and Use Committee of the First Affiliated Hospital of Zhengzhou University and conducted in accordance with the NIH Guide for the Care and Use of Laboratory Animals. ChAT‐IRES‐Cre mice on a C57BL/6J background were provided by Dr. Cheng Xiao (Xuzhou Medical University). The propagation of ChAT‐IRES‐Cre mice via in vitro fertilization (IVF) was facilitated by Shanghai Model Organism Center Inc. (Shanghai, China). Male C57BL/6 mice aged 18–20 months were acquired from Junke Biological Co. (Nanjing, China). Mice were housed under a 12‐h light/dark cycle with *ad libitum* access to food and water. Every effort was made to minimize the number of animals used in the study.

### 2.2 Exploratory Laparotomy

The laparotomy procedure was performed as previously described (Jia et al. 2025). Briefly, after 4–6 h of fasting, mice were anesthetized with isoflurane (3% induction, 1%–2% maintenance). The gas concentration within the anesthesia chamber was continuously monitored using a multiparametric anesthetic gas monitor. Surgery group animals received the same oxygen concentration (30%) during isoflurane anesthesia. Under sterile conditions, a 1‐cm midline abdominal incision was made, and the small intestine was exteriorized and exposed for 10 min before being returned to the abdominal cavity. Care was taken to ensure that the small intestine was covered with wet gauze and maintained in the same orientation upon reinsertion into the abdominal cavity. The muscle fascia and skin were sutured, and 0.2% ropivacaine was administered for analgesia. The entire procedure lasted approximately 30 min. Following the surgery, the mice were allowed to recover on a heating pad until they regained consciousness and were subsequently returned to their home cages. Control animals were exposed to 30% oxygen for 30 min without anesthesia or surgery. Thus, both groups were exposed to 30% oxygen for approximately 30 min, ensuring comparable oxygenation conditions.

### 2.3 Behavioral Tests

A well‐trained investigator blinded to the animal groups conducted all behavioral tests during the light phase (09:30–14:00).

Open Field Test (OFT): Mice were placed in a square box (45 × 45 × 45 cm) and allowed to explore freely for 10 min. Their movements were recorded and analyzed using Panlab SMART 3.0 software (RWD Life Science, Shenzhen, China). The total distance traveled and the percentage of time spent in the central area during the last 5 min were calculated.

Novel Object Recognition (NOR): The test was conducted in a black plastic box (60 × 40 × 80 cm). During the familiarization phase, mice explored two identical objects for 10 min. After a 24 h interval, one object was replaced with a novel one, and exploration times were recorded. The discrimination index (DI) was calculated as ((N‐T)/(*N* + T)) × 100.

Fear Conditioning (FC) Test: During training, mice were placed in a conditioning chamber and received four tone‐shock pairings (90 dB tone, 20 s; 0.2 mA foot shock during the last 2 s). After 24 h, contextual fear memory was assessed by returning the mice to the original training chamber for 5 min, and freezing behavior was quantified. 2 h later, cued fear memory was assessed by placing the mice in a novel context (with distinct flooring, wall patterns, and odor) and presenting the conditioned tone for 3 min; freezing behavior was measured during tone presentation. Freezing was defined as the absence of all movement except respiration. Behavior was recorded by a video camera and analyzed using the VisuFcs condition fear conditioning analysis system (XR‐XC404; Shanghai Xinruan, Shanghai, China). Freezing was quantified as the percentage of time spent freezing.

### 2.4 Long‐Term Potentiation (LTP) Recording

Hippocampal slices (300 μm) were prepared and maintained in artificial cerebrospinal fluid (ACSF). Field excitatory postsynaptic potentials (fEPSPs) were recorded in the CA1 region following Schaffer collateral stimulation. LTP was induced by high‐frequency stimulation (HFS; 100 Hz tetanic stimulation) consisting of three trains of 100 pulses at 100 Hz with a 10 s inter‐train interval. The magnitude of LTP was determined by averaging fEPSP slopes 50–60 min post‐induction.

### 2.5 Viral Vectors and Drug

The following viral vectors were used: rAAV‐hSyn‐DIO‐GCaMP6s‐WPREs, rAAV‐hSyn‐DIO‐mCherry‐WPRE‐hGH polyA, rAAV‐hSyn‐DIO‐hM3D(Gq)‐mCherry‐WPRE‐hGH polyA (Brain VTA, China), pRetro‐U6‐EF1‐EGFP‐3xFLAG‐WPRE (OBiO, China), and AAV2/9‐hSyn‐ACh3.0 (WZ Biosciences Inc., China). All viral vectors had titers ≥ 2 × 10^12^ vg/mL. Acetylcholinesterase inhibitor galantamine was acquired from Suicheng Pharmaceutical Co. (Zhengzhou, China).

### 2.6 Virus Injection and Cannula Implantation

Mice were anesthetized with isoflurane and placed in a stereotaxic apparatus (RWD Life Science, Shenzhen, China). A homeothermic heating blanket was set at 37°C to maintain the body temperature of the mice throughout the surgical and experimental procedures. Viruses were injected into the MS/vDB or hippocampus at a rate of 20–50 nL/min. For the MS/vDB, coordinates were: AP = +0.50 mm, ML = ±0.45 mm, DV = −4.60 mm; 200 nL per side. For the DG: AP = −2.00 mm, ML = ±1.40 mm, DV = −2.10 mm; 300 nL per side. Given mild brain atrophy in 18‐month‐old mice, DV coordinates were adjusted 0.1–0.2 mm downward relative to young adult mice. All coordinates were based on Paxinos and Franklin (4th edition). For pharmacological manipulation, guide cannulas (RWD Life Science, Shenzhen, China) were implanted 100 μm above the viral injection site. Mice subjected to viral injection were allowed to recover for at least 3 weeks before experiments. Mice subjected to cannula implantation were allowed to recover for at least 1 week.

### 2.7 Fiber Photometry Recording and Analysis

For fiber photometry, rAAV‐hSyn‐DIO‐GCaMP6s was injected into the MS/vDB and ACh3.0 into the DG. The coordinates were described above. An optical fiber was implanted 100 μm above the viral delivery site. Fluorescence signals were recorded using a fiber photometry system (RWD Life Science, China). The ΔF/F ratio was calculated as (470 nm signal–fitted 410 nm signal)/fitted 410 nm signal.

### 2.8 Chemogenetic Manipulation and Pharmacological Modulation

For chemogenetic activation, rAAV‐hSyn‐DIO‐hM3D(Gq)‐mCherry or control virus was injected into the MS/vDB. Clozapine‐N‐oxide (CNO; 5 μM/0.2 μL/side) or MEC (10 μM/0.2 μL/side) was bilaterally injected into the DG 45 or 15 min before behavioral testing, respectively. For chronic activation, CNO was administered daily for 14 days.

### 2.9 Immunohistochemistry

Mice were perfused with PBS followed by 4% paraformaldehyde. Brains were sectioned (30 μm) using a cryostat (VT‐1200S, Leica, Germany) and stained with primary antibodies against c‐Fos (ab190289, Abcam, diluted 1:1000), ChAT (AB143, Sigma, diluted 1:500), DCX (13925–1‐AP, Proteintech, diluted 1:500), DCX (sc‐8066, Santa Cruz, diluted 1:500), ɑ7 nAChR (ANC‐007‐FR, Alomone Labs, diluted 1:100), and BrdU (ab6326, Abcam, diluted 1:500). Secondary antibodies were Anti‐Rat Alexa Fluor 488, Anti‐Goat Cy3, and Anti‐Rabbit Cy3 (Jackson ImmunoResearch, diluted 1:400). Images were acquired using a confocal microscope (Olympus Fv‐1000, Japan).

### 2.10 Genotyping of Transgenic Mice

Genomic DNA was isolated from tail clips and genotyped by PCR using specific primers. The expected band sizes were: mutant (350 bp), heterozygote (350 bp and 272 bp), and wild type (272 bp). The primers used were: Primer 1 (Mutant Reverse): 5′‐CCTTCTATCGCCTTCTTGACG‐3′; Primer 2 (Common): 5′‐AGATAGATAATGAGAGGCTC‐3′; Primer 3 (Wild type Reverse): 5′‐GTTTGCAGAAGCGGTGGG‐3′.

### 2.11 Quantifications and Sholl Analysis

Image analyses were performed blinded using ImageJ (Fiji). The Cell Counter plugin was used for cell counting. Sholl analysis was conducted by counting dendritic intersections with concentric circles (25 μm increments). Total dendritic length and branch number were also quantified.

### 2.12 Statistical Analysis

Data are presented as mean ± SEM. Statistical analyses were performed using GraphPad Prism v7.0. Unpaired *t*‐tests or Mann–Whitney *U* tests were used for two‐group comparisons. One‐way ANOVA followed by Tukey's test was used for multiple comparisons. Two‐way ANOVA with Tukey's post hoc test was applied to assess the interaction between surgery and cholinergic interventions. *p* < 0.05 was considered statistically significant.

## 3 Results

### 3.1 Surgery Induces Cognitive Deficits, Synaptic Plasticity Impairment, and Reduces AHN in Aged Mice

To investigate the impact of anesthesia and surgery on behavioral phenotypes, we employed an exploratory laparotomy model of POCD in aged mice. Arterial blood gas analysis confirmed comparable oxygenation between surgery and control groups (Figure S1), excluding differential oxygen exposure as a confounding factor. In the OFT, surgically treated mice exhibited normal locomotor activity (Figure S2C) but spent significantly less time in the center zone (Figure S2D), indicating increased anxiety‐like behavior in this paradigm. In the NOR test, surgery significantly reduced the DI (Figure S2E–H), reflecting impaired recognition memory. In the fear conditioning test, surgery markedly decreased freezing behavior in the cued but not contextual memory test (Figure S2I–K). Electrophysiological recordings on postoperative Day 9 revealed a significant impairment in hippocampal LTP (Figure S3), suggesting compromised synaptic plasticity.

**FIGURE 1:**
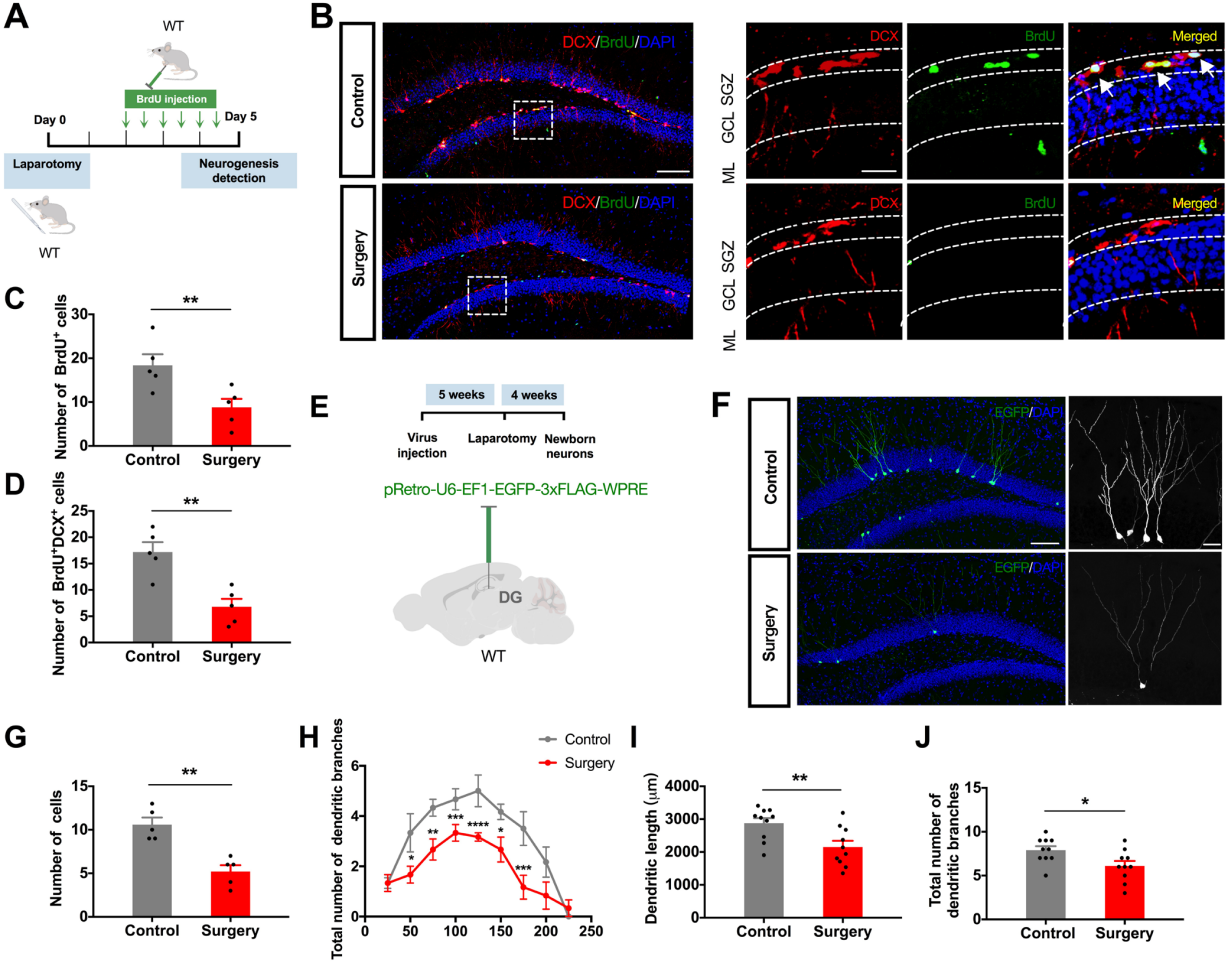
Surgery suppresses adult hippocampal neurogenesis and impairs dendritic maturation in aged mice. (A) Schematic of the experimental design for assessing postoperative AHN and neuronal morphology. (B) Representative confocal images of DCX^+^BrdU^+^ cells (solid arrows) in the SGZ. Scale bars: 100 μm (overview) and 20 μm (insets). (C, D) Quantification of BrdU^+^ cells (unpaired *t*‐test, *t*(8) = 3.036, *p* = 0.016) (C) and DCX^+^BrdU^+^ cells (unpaired *t*‐test, *t*(8) = 4.326, *p* = 0.003) (D) in the DG. *N* = 5 mice per group. (E) Schematic of the experimental design for assessing postoperative neuronal morphology. (F) Representative confocal images of EGFP^+^ newborn neurons in the DG (left) and high‐magnification images of individual neurons (right). Scale bars: 100 μm (overview) and 50 μm (single neurons). (G) Density of EGFP^+^ newborn neurons (unpaired *t*‐test, *t*(8) = 4.93, *p* = 0.001). *N* = 5 mice per group. (H) Sholl analysis of dendritic complexity (Two‐way ANOVA, row factor: *F*(8, 90) = 17.86, *p* < 0.0001; column factor: *F*(1, 90) = 35.35, *p* < 0.0001). (I, J) Quantification of total dendritic length (unpaired *t*‐test, *t*(18) = 2.954, *p* = 0.009) (I) and total dendritic branches (unpaired *t*‐test, *t*(18) = 2.470, *p* = 0.024) (J) per neuron. *N* = 10 neurons per group (from 5 mice). Data are presented as mean ± SEM. *p < 0.05, **p < 0.01, ***p < 0.001, ****p < 0.0001.

Given the crucial role of AHN in cognitive function, we next assessed its status (Figure 1A). Immunohistochemistry showed that surgery significantly reduced the number of BrdU^+^ and DCX^+^BrdU^+^ cells in the SGZ of aged mice (Figure 1B–D), indicating suppressed proliferation and neuronal differentiation. Using a retrovirus‐based labeling approach (Figure 1E), we further demonstrated that surgery led to a significant simplification of neuronal architecture, characterized by reduced dendritic length and branching complexity in new neurons (Figure 1F–J). Collectively, these findings establish that surgery in aged mice results in a broad spectrum of hippocampal dysfunction, encompassing anxiety‐like behavior, memory deficits, impaired synaptic plasticity, and substantial impairment of AHN.

### 3.2 Surgery Attenuates ACh Release in the DG of Aged Mice

**FIGURE 2:**
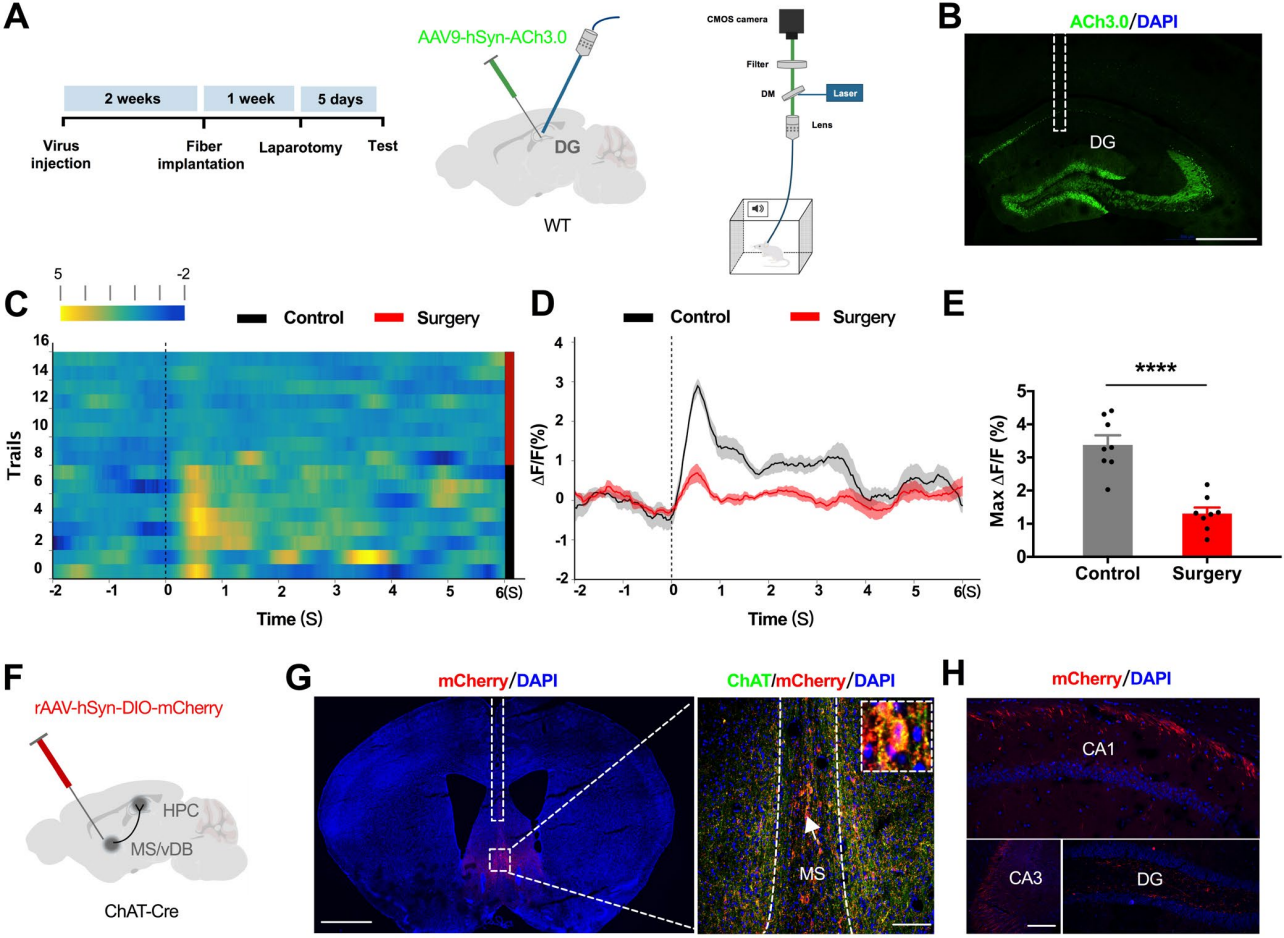
Surgery reduces acetylcholine release from MS/vDB terminals in the DG. (A) Schematic of intra‐DG injection of AAV9‐hSyn‐ACh3.0 and optical fiber implantation for fiber photometry recording of ACh release. (B) Representative image showing optical fiber placement in the DG. Scale bar: 500 μm. (C‐E) ACh signals in the DG evoked by a 1 s auditory cue during FC, presented as a heatmap (C), trace (D), and quantified amplitude (E) (unpaired *t*‐test, *t*(14) = 6.109, *p* < 0.0001). *N* = 8 mice per group. (F) Viral strategy for anterograde tracing of MS/vDB → DG projections: RAAV‐hSyn‐DIO‐mCherry injected into the MS/vDB of ChAT‐Cre mice. (G) Representative images showing mCherry expression in ChAT^+^ neurons in the MS/vDB. Dashed line indicates fiber track. Scale bars: 1000 μm (left) and 100 μm (right). (H) Representative images of mCherry‐labeled cholinergic projections in hippocampal CA1, CA3, and DG. Scale bar: 200 μm. Data are presented as mean ± SEM. ***p < 0.0001.

We next sought to determine if these neurogenic deficits were associated with alterations in cholinergic signaling. Immunofluorescence staining revealed that α7 nAChRs are expressed on a subset of BrdU^+^ and DCX^+^ cells in the DG of aged mice (Figure S4), suggesting a potential direct modulation of AHN by cholinergic inputs via nAChRs. To monitor real‐time ACh dynamics in the DG, we expressed the genetically encoded ACh sensor ACh 3.0 and implanted an optical fiber above the DG (Figure 2A). Three weeks post‐viral infection, correct EGFP expression and fiber placement in the DG were confirmed (Figure 2B). During a cued fear conditioning paradigm, the ACh release evoked by the auditory cue was significantly attenuated in surgery mice compared with controls (Figure 2C–E). Since the MS/vDB provides the major cholinergic input to the hippocampus (Wu et al. 2024), we then mapped these projections by injecting a Cre‐dependent rAAV‐hSyn‐DIO‐mCherry into the MS/vDB of ChAT‐Cre mice (Figure 2F). Viral tracing confirmed the presence of dense mCherry^+^ cholinergic projections throughout the hippocampus, including the CA1, CA3, and DG (Figure 2G,H). These data suggest that the reduced ACh levels in the DG after surgery likely originate from a decrease in upstream cholinergic input.

### 3.3 Surgery Suppresses the Activity of MS/vDB Cholinergic Neurons in Aged Mice

**FIGURE 3:**
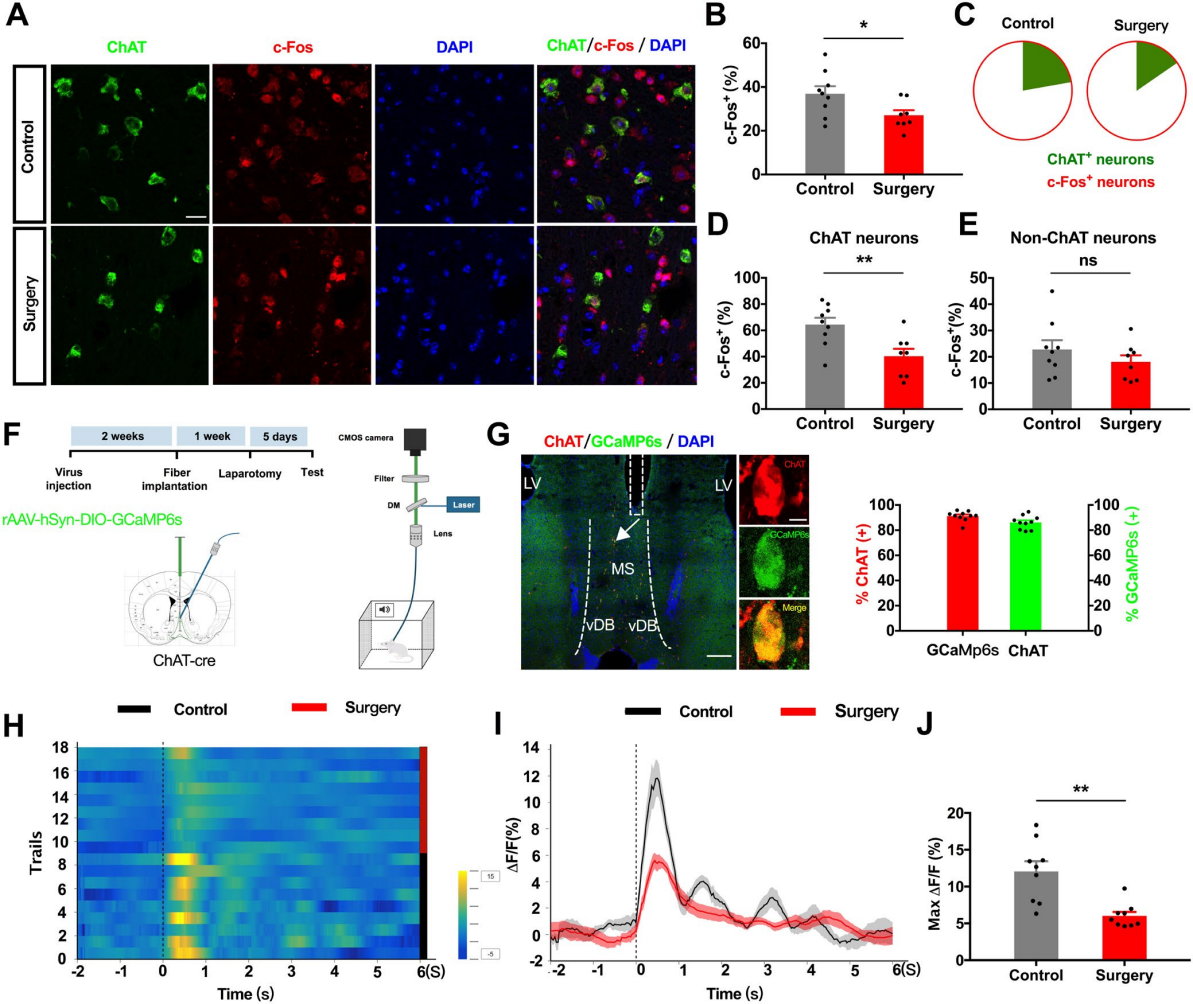
Surgery dampens calcium activity in MS/vDB cholinergic neurons. (A) Representative images of c‐Fos and ChAT expression in the MS/vDB. Scale bar: 20 μm. (B–E) Quantification of c‐Fos^+^/DAPI^+^ cells (Unpaired *t*‐test, *t*(15) = 2.34, *p* = 0.034) (B), ChAT^+^c‐Fos^+^/c‐Fos^+^ in control mice (left) and ChAT^+^c‐Fos^+^/c‐Fos^+^ in surgery mice (right) (C), ChAT^+^c‐Fos^+^/ChAT^+^ (Unpaired *t*‐test, *t*(15) = 3.124, *p* = 0.007) (D), and ChAT^−^c‐Fos^+^ /ChAT^−^ (Unpaired *t*‐test, *t*(15) = 1.07, *p* = 0.302) (E) cells in the MS/vDB. *N* = 8–9 sections per group from 3 mice. (F) Experimental schematic: RAAV‐hSyn‐DIO‐GCaMP6s injected into MS/vDB, fiber photometry recordings during a 10‐min FC session. (G) Representative image of fiber placement in MS/vDB. Scale bars: 200 μm (left) and 5 μm (right). (H, I) Heatmap (H) and trace (I) of calcium signals evoked by 1 s auditory cue. (J) Quantification of area under the curve (AUC) shown in (H) (Mann–Whitney test, *U* = 6, *p* = 0.001). *N* = 9 mice per group. Data are presented as mean ± SEM. *p < 0.05, **p < 0.01; ns, not significant.

To directly evaluate the activity of upstream cholinergic neurons, we examined c‐Fos expression, a marker of neuronal activation, in the MS/vDB. Surgery significantly decreased the number of c‐Fos^+^ neurons, and this reduction was particularly prominent within ChAT^+^ cholinergic neurons (Figure 3A–E). To gain dynamic insight into their activity, we expressed the Cre‐dependent calcium indicator GCaMP6s in the MS/vDB of ChAT‐Cre mice and recorded calcium transients via an implanted fiber (Figure 3F). Histological analysis confirmed that over 90% of GCaMP6s‐labeled cells were ChAT^+^, and approximately 85% of ChAT neurons expressed GCaMP6s (Figure 3G). During exposure to the FC test, control mice exhibited a robust increase in Ca^2+^ signals in the MS/vDB, whereas this response was significantly blunted in surgery mice (Figure 3H–J). These results indicate that MS/vDB cholinergic neurons in aged mice display hypo‐responsiveness during cognitive tasks following anesthesia and surgery.

### 3.4 Enhancing Cholinergic Tone in the DG Rescues Memory Deficits and Restores AHN

**FIGURE 4:**
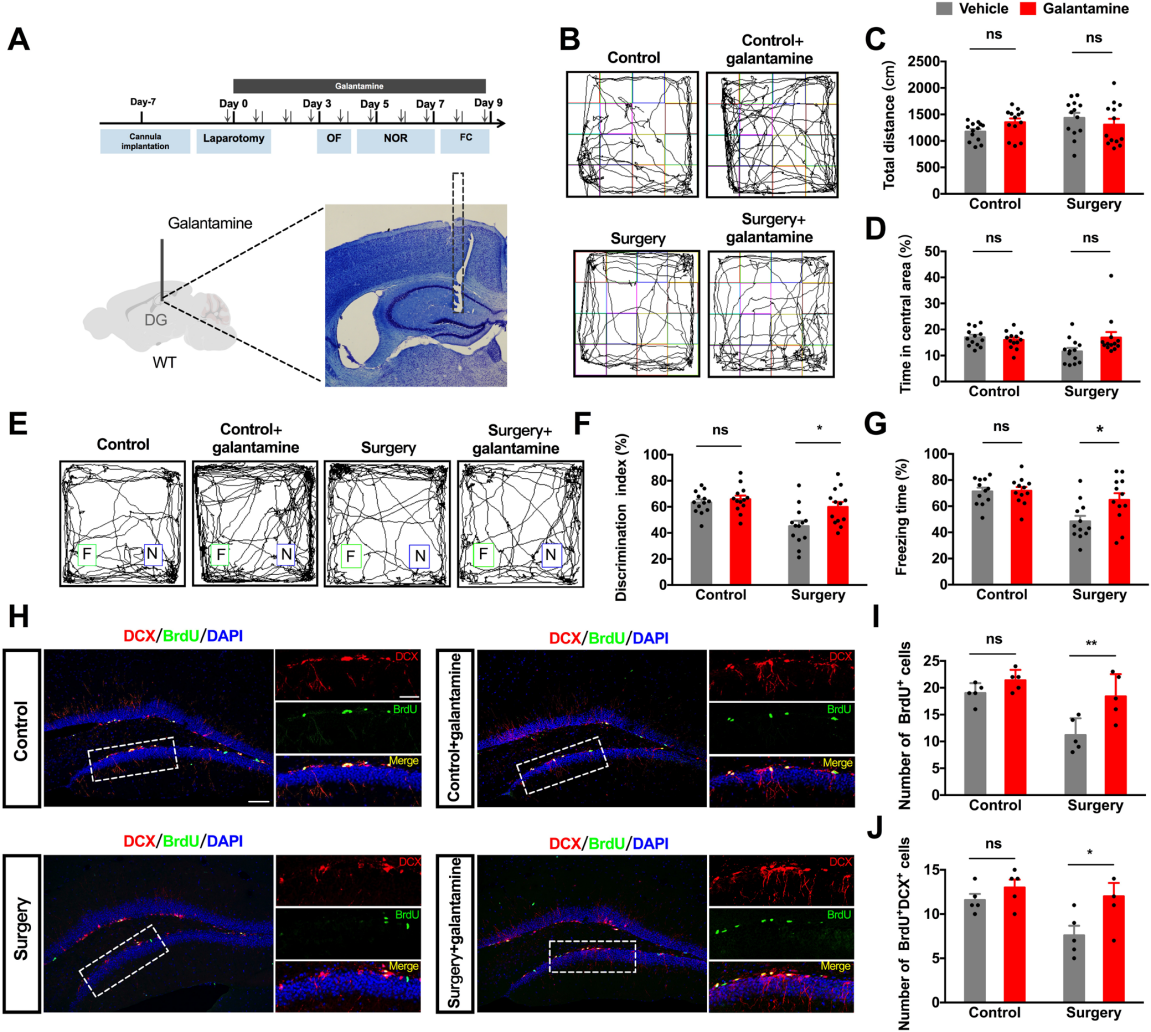
Chronic galantamine infusion specifically rescues surgery‐induced memory deficits and AHN impairment. Schematic of drug administration protocol: Bilateral intra‐DG microinfusions of galantamine (10 μM/0.2 μL/side) or saline (0.2 μL/side) for 9 consecutive days. Dashed line indicates cannula placement in DG. (B) Representative movement tracks in the OFT. (C, D) Quantification of total distance (C) and center time percentage (D). Two‐way ANOVA revealed a significant surgery × galantamine interaction for center time (*F*(1,48) = 4.988, *p* = 0.030). Post hoc comparisons: Surgery‐vehicle mice spent less center time than control‐vehicle mice (*p* = 0.044); galantamine had no effect in controls (*p* = 0.957) and showed a trend toward improvement in surgery mice (*p* = 0.051). No effect on total distance. *N* = 13 per group. (E) Representative NOR exploration tracks. (F) DI in NOR. Two‐way ANOVA: Significant main effects of galantamine (*F*(1,44) = 13.05, *p* = 0.001) and surgery (*F*(1,44) = 6.480, *p* = 0.013); interaction not significant (*p* = 0.077). Post hoc: Galantamine increased DI in surgery mice (*p* = 0.017) but not in controls (*p* = 0.950). *N* = 12 per group. (G) Freezing percentage in the cued FC. Two‐way ANOVA: Significant surgery × galantamine interaction (*F*(1,44) = 4.138, *p* = 0.048). Post hoc tests: Galantamine increased freezing in surgery mice (*p* = 0.024) but not in controls (*p* = 1.000). *N* = 11–12 per group. (H) Representative confocal images of DCX^+^BrdU^+^ cells in SGZ. Scale bars: 100 μm (overview) and 20 μm (insets). (I) Quantification of BrdU^+^ cells. Two‐way ANOVA: Significant main effects of surgery (*F*(1,16) = 17, *p* = 0.001) and galantamine (*F*(1,16) = 13.43, *p* = 0.002); interaction not significant (*F*(1,16) = 3.359, *p* = 0.086). Post hoc: Galantamine increased BrdU^+^ cells in surgery mice (*p* = 0.006) but not in controls (*p* = 0.578). (J) Quantification of DCX^+^BrdU^+^ cells. Two‐way ANOVA: Significant main effects of surgery (*F*(1,16) = 5.297, *p* = 0.035) and galantamine (*F*(1,16) = 7.127, *p* = 0.017); interaction not significant (*F*(1,16) = 1.907, *p* = 0.186). Post hoc: Galantamine increased DCX^+^BrdU^+^ cells in surgery mice (*p* = 0.050) but not in controls (*p* = 0.799). Data are presented as mean ± SEM. *p < 0.05, **p < 0.01; ns, not significant.

We hypothesized that elevating ACh levels in the DG could mitigate postoperative cognitive deficits. Starting from the day of surgery, we infused the acetylcholinesterase inhibitor galantamine (10 μM/0.2 μL/side) or saline bilaterally into the DG for nine consecutive days (Figure 4A). OFT on postoperative Day 3 showed no differences in total distance traveled (Figure 4B,C), excluding gross motor effects. Galantamine did not affect center time in control mice but showed a trend toward improvement in surgery mice (Figure 4D). In the NOR test, galantamine significantly increased the DI in surgery mice but had no effect in controls (Figure 4E,F). Similarly, in the cued FC test, galantamine significantly elevated freezing time in surgery mice but not in controls (Figure 4G). In a separate cohort, galantamine significantly increased the number of DCX^+^/BrdU^+^ cells in surgery mice, with no effect in controls (Figure 4H–J). Collectively, these findings demonstrate that chronic galantamine infusion in the DG specifically ameliorates surgery‐induced memory deficits and AHN impairment, with no significant effects under baseline physiological conditions in non‐surgery aged mice.

### 3.5 Acute Chemogenetic Activation of MS/vDB→DG Cholinergic Projections Alleviates Anxiety‐Like Behavior but Not Memory Deficits

**FIGURE 5:**
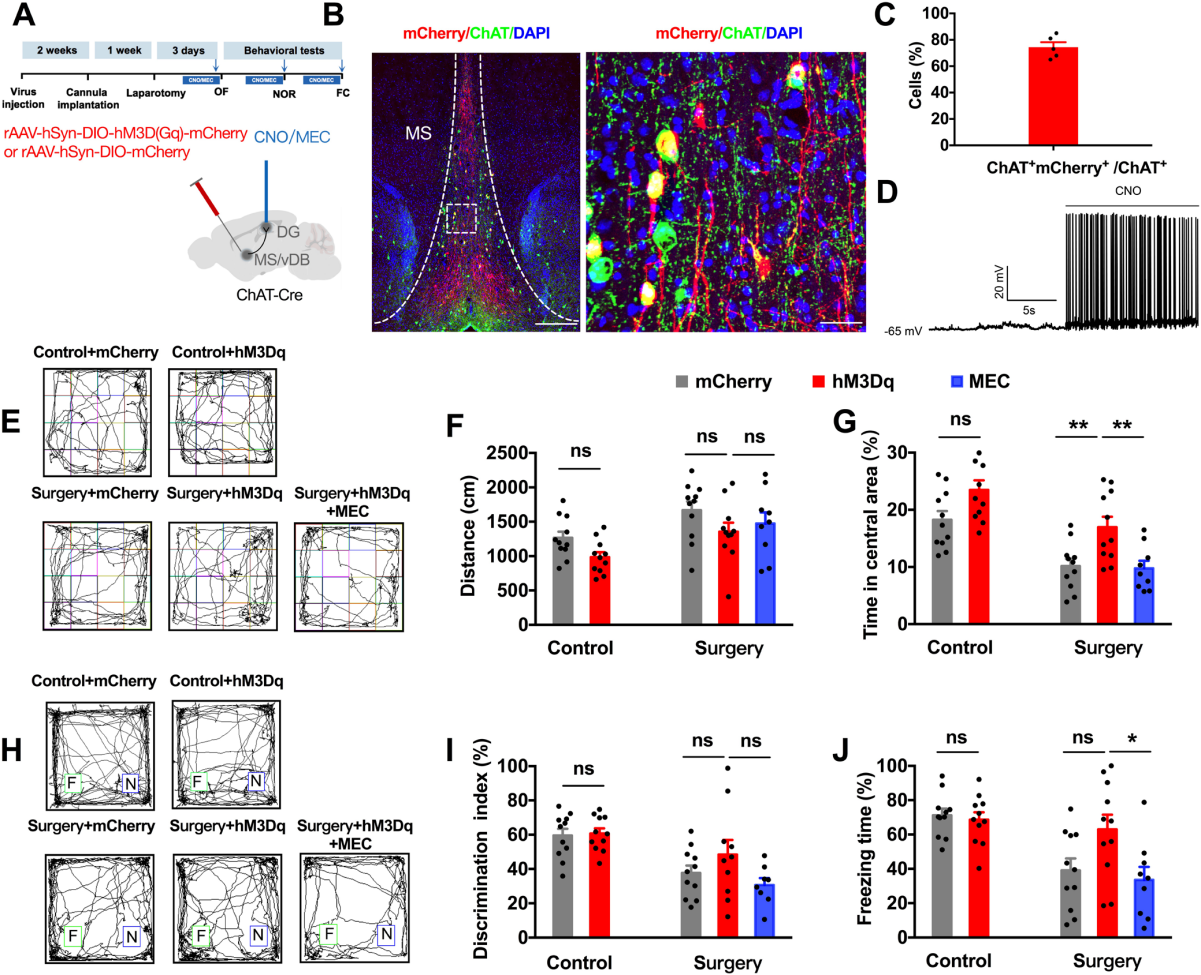
Acute chemogenetic activation of MS/vDB→DG cholinergic projections reduces anxiety‐like behavior but does not rescue memory deficits. Schematic of experimental design: RAAV‐hSyn‐DIO‐hM3D(Gq)‐mCherry or control rAAV‐hSyn‐DIO‐mCherry injected into MS/vDB of ChAT‐Cre mice. CNO (5 μM) or MEC (10 μM) microinfused into bilateral DG before behavioral tests. (B, C) Representative images of hM3D(Gq)‐mCherry (red) colocalized with ChAT^+^ neurons (green) in MS/vDB. Scale bars: 100 μm (left) and 20 μm (right). (D) Current–voltage (I‐V) relationships of a representative MS/vDB neuron before and during perfusion with 5 μM CNO, confirming DREADD activation. (E–G) OFT. (E) Representative movement traces. (F) Total distance: Two‐way ANOVA showed significant main effects of surgery (*F*(1,40) = 12.56, *p* = 0.001) and hM3Dq (*F*(1,40) = 7.32, *p* = 0.010), no interaction (*F*(1,40) = 0.021, *p* = 0.886); post hoc pairwise comparisons not significant (all adjusted *p* > 0.05). (G) Center time: Two‐way ANOVA showed main effects of surgery (*F*(1,40) = 21.23, *p* < 0.0001) and hM3Dq (*F*(1,40) = 14.36, *p* = 0.001), no interaction (*F*(1,40) = 0.240, *p* = 0.627). Post hoc: HM3Dq increased center time in surgery mice (*p* = 0.022), but not controls (*p* = 0.107). In surgery mice, MEC co‐administration blocked this effect (one‐way ANOVA among surgery groups: *F*(2,21) = 7.26, *p* = 0.003; post hoc: HM3Dq + MEC vs. hM3Dq, *p* = 0.014). (H, I) NOR. (H) Representative exploration paths. (I) DI: Two‐way ANOVA showed a main effect of surgery (*F*(1,39) = 10.63, *p* = 0.002) but no effect of hM3Dq (*F*(1,39) = 1.292, *p* = 0.263) and no interaction (*F*(1,39) = 0.839, *p* = 0.365). Post hoc: HM3Dq did not improve DI in surgery mice (*p* = 0.486) nor controls (*p* = 0.999). (J) Cued FC. Two‐way ANOVA: Main effect of surgery (*F*(1,40) = 8.293, *p* = 0.006) but no significant effect of hM3Dq (*F*(1,40) = 3.044, *p* = 0.089) and a trend toward interaction (*F*(1,40) = 4.034, *p* = 0.051). Post hoc: HM3Dq did not improve freezing in surgery mice (*p* = 0.053) nor controls (*p* = 0.998). Data are presented as mean ± SEM. *N* = 8–11 mice per group. *p < 0.05, **p < 0.01; ns, not significant.

We next examined whether direct activation of the MS/vDB → DG cholinergic pathway confers behavioral benefits. The excitatory DREADD hM3Dq or mCherry control was expressed in the MS/vDB of ChAT‐Cre mice. CNO (5 μM/0.2 μL/side) was infused into the DG 45 min before behavioral tests. To assess whether the effects of chemogenetic activation require nAChR signaling, we co‐administered the nAChR antagonist MEC (10 μM/0.2 μL/side) or saline (0.2 μL/side) 15 min prior (Figure 5A). nAChRs were specifically targeted given their established roles in adult hippocampal neurogenesis and cholinergic modulation of DG function (Campbell et al. 2010; Mukhtar et al. 2025). Immunostaining confirmed that approximately 74.4% ± 3.8% of ChAT^+^ neurons were successfully labeled with mCherry (Figure 5B,C), and electrophysiological recordings verified that CNO effectively activated MS/vDB ChAT neurons (Figure 5D).

In the OFT, acute CNO‐induced activation of MS/vDB → DG projections did not alter locomotor activity (Figure 5E,F). Acute activation increased center time in surgery mice, and this effect was blocked by MEC, confirming nAChR dependence (Figure 5G). In non‐surgery mice, acute activation did not significantly alter center time, suggesting that the anxiolytic‐like effect of acute activation may be more pronounced in the context of surgical injury (Figure 5G). By contrast, acute activation failed to rescue surgery‐induced deficits in either NOR (Figure 5H,I) or cued FC (Figure 5J). Moreover, it did not improve memory performance in non‐surgery mice (Figure 5H–J). Thus, acute MS/vDB → DG cholinergic activation increases OFT center time in an nAChR‐dependent manner, an effect significant only in surgery mice but fails to rescue memory deficits, indicating that sustained signaling is required for cognitive recovery.

### 3.6 Sustained Chemogenetic Activation of MS/vDB → DG Cholinergic Signaling Rescues Memory and Promotes Neurogenesis

**FIGURE 6:**
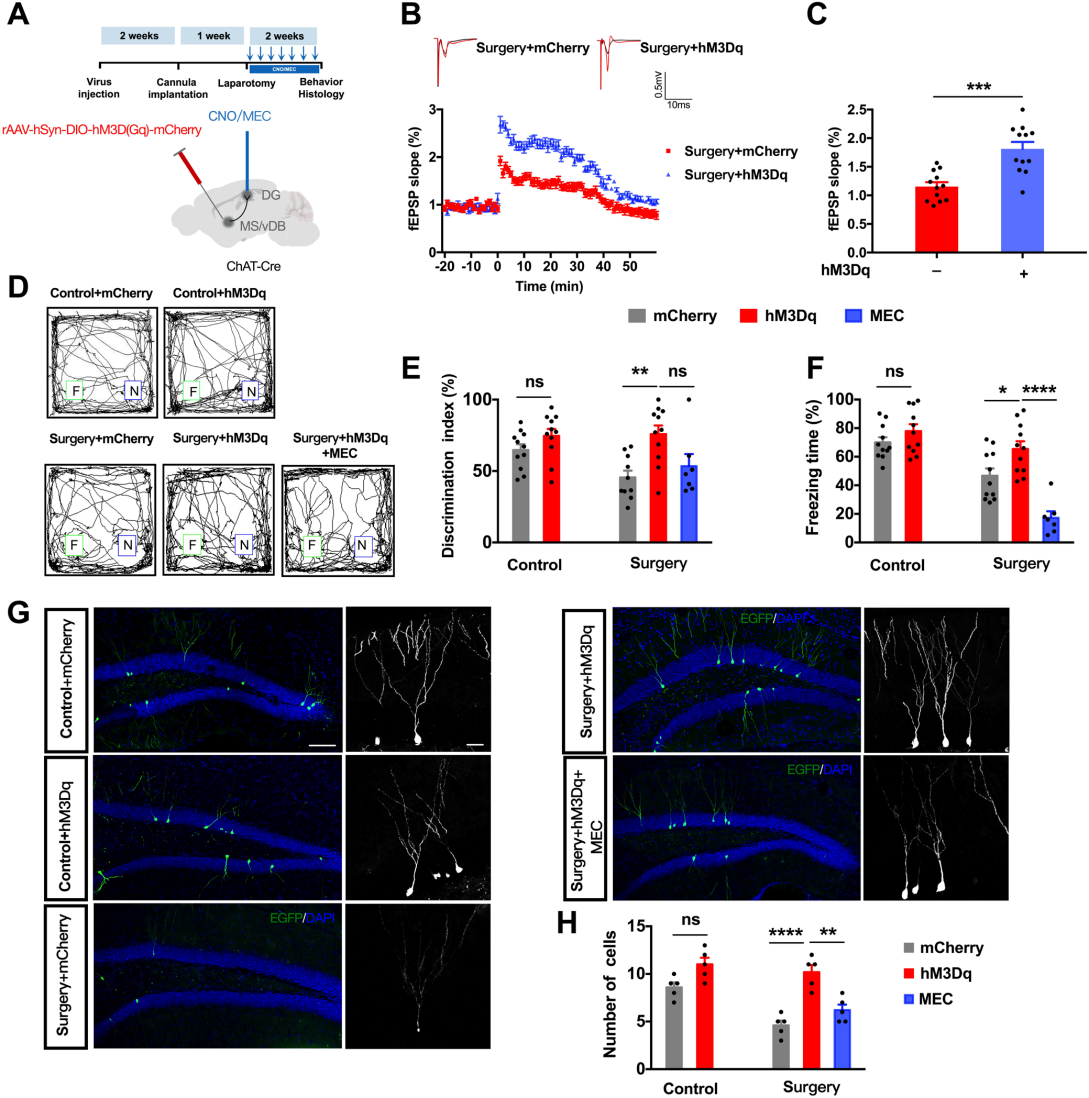
Sustained chemogenetic activation of MS/vDB→DG cholinergic projections rescues memory deficits and promotes AHN. Schematic of sustained chemogenetic activation strategy: ChAT‐Cre mice received bilateral MS/vDB injections of hM3Dq or mCherry virus; intra‐DG CNO (or CNO + MEC) microinfusions for 14 consecutive days. (B, C) LTP at hippocampal Schaffer collateral→CA1 synapses induced by 100 Hz tetanic stimulation. (B) Representative fEPSP traces. (C) Quantification of LTP levels (unpaired *t*‐test, *t*(22) = 4.183, *p* < 0.001). *N* = 12 slices from 4 mice per group. (D–F) Behavioral assessments. (D) Representative NOR exploration tracks. (E) DI in NOR: Two‐way ANOVA revealed significant interaction (*F*(1,39) = 4.166, *p* = 0.048), main effect of hM3Dq (*F*(1,39) = 16.16, *p* < 0.001), and non‐significant main effect of surgery (*F*(1,39) = 3.254, *p* = 0.079). Post hoc: Chronic activation increased DI in surgery mice (*p* = 0.001) but not in controls (*p* = 0.497). In surgery mice, MEC blocked this effect (one‐way ANOVA among surgery groups: *F*(2,22) = 8.12, *p* = 0.002; post hoc: HM3Dq + MEC vs. hM3Dq, *p* = 0.014). (F) Freezing in cued FC: Two‐way ANOVA showed significant interaction (*F*(1,40) = 4.165, *p* = 0.048), main effects of surgery (*F*(1,40) = 14.92, *p* < 0.001) and hM3Dq (*F*(1,40) = 5.285, *p* = 0.027). Post hoc: Chronic activation increased freezing in surgery mice (*p* = 0.019) but not in controls (*p* = 0.998). MEC attenuated this rescue (one‐way ANOVA: *F*(2,21) = 9.45, *p* = 0.001; post hoc: HM3Dq + MEC vs. hM3Dq, *p* = 0.008). *N* = 7–11 per group. (G, H) AHN. (G) Representative confocal images of EGFP^+^ newborn neurons in DG (left, scale bar: 100 μm) and high‐magnification images (right, scale bar: 50 μm). (H) Density of EGFP^+^ neurons: Two‐way ANOVA showed significant interaction (*F*(1,16) = 6.564, *p* = 0.021), main effects of surgery (*F*(1,16) = 14.77, *p* = 0.001) and hM3Dq (*F*(1,16) = 41.03, *p* < 0.0001). Post hoc: Chronic activation increased neurogenesis in surgery mice (*p* < 0.0001) but not in controls (*p* = 0.066). MEC prevented this increase (one‐way ANOVA: *F*(2,12) = 15.67, *p* < 0.001; post hoc: HM3Dq + MEC vs. hM3Dq, *p* = 0.002). *N* = 5 mice per group. Data are presented as mean ± SEM. *p < 0.05, **p < 0.01, ***p < 0.001, ****p < 0.0001; ns, not significant.

Given that lasting memory improvements likely require sustained plasticity, we prolonged pathway activation. After viral expression, mice received intra‐DG CNO infusions for 14 days, with or without MEC co‐administration (Figure 6A). Sustained activation significantly enhanced LTP at hippocampal Schaffer collateral‐CA1 synapses compared with mCherry controls (Figure 6B,C), indicating improved synaptic plasticity. In the NOR test, chronic activation significantly increased the DI in surgery mice but not in controls (Figure 6D,E). Similarly, in cued FC, chronic activation significantly elevated freezing time in surgery mice but not in controls, and this effect was blocked by MEC (Figure 6F). Unlike acute activation, chronic activation did not alter center time in the OFT (Figure S5), indicating that the anxiolytic effect of acute activation is not sustained with prolonged stimulation.

We next assessed AHN. Four weeks after surgery, chronic activation significantly increased the number of newborn neurons in surgery mice, whereas the increase in control mice did not reach statistical significance; MEC co‐administration prevented this increase (Figure 6G,H). Collectively, these results demonstrate that sustained, but not acute, activation of MS/vDB → DG cholinergic signaling rescues postoperative memory deficits and promotes AHN through nAChR‐dependent mechanisms, with effects specifically amplified by the surgical insult.

## 4 Discussion

This study provides compelling evidence that dysfunction of the septohippocampal cholinergic signaling underlies surgery‐induced cognitive and neurogenic impairments in aged mice. We demonstrate that exploratory laparotomy in aged mice leads to three key deficits: (1) substantial impairment of AHN, evidenced by reduced proliferation and maturation of newborn neurons in the SGZ; (2) attenuated ACh release in the DG during cognitive tasks; and (3) hypoactivity of MS/vDB cholinergic neurons—the primary source of hippocampal cholinergic input. Crucially, restoring cholinergic tone—either pharmacologically with the acetylcholinesterase inhibitor galantamine or via sustained chemogenetic activation of MS/vDB → DG projections—significantly rescues both deficits, with additional control experiments confirming these effects are specific to surgery‐induced deficits rather than representing a general cognitive or neurogenic enhancement.

Using a highly sensitive genetically encoded ACh sensor, we detected attenuated ACh release in the hippocampus of surgically treated mice during cognitive tasks. Given the established role of ACh in modulating synaptic plasticity and memory (Hasselmo 2006), we hypothesized boosting cholinergic signaling could mitigate postoperative deficits. Local intra‐DG galantamine administration ameliorated memory impairments and restored AHN, while the same regimen failed to improve NOR or FC performance, nor did it increase neurogenesis in non‐surgery aged mice. These findings indicate that the beneficial effects of galantamine are specifically attributable to the reversal of surgery‐induced cholinergic dysfunction.

The hippocampus maintains the unique capacity to generate new neurons throughout adulthood, a process closely linked to brain plasticity, learning, and repair (Salta et al. 2023; Ming and Song 2011; Li, Luo, et al. 2022). Notably, the proliferative capacity of neural stem cells (NSCs) declines sharply with age and is further compromised by surgical inflammation and stress (Encinas et al. 2011; Barrientos et al. 2012), underscoring the importance of identifying strategies to preserve the NSC pool. We discovered that surgery in aged mice impairs cell proliferation (reduced BrdU^+^ cells), indicating that surgical stress strongly suppresses the proliferative activity of stem/precursor cells in the DG. Furthermore, we observed a reduction in neuronal differentiation of newborn cells (decreased BrdU^+^DCX^+^ cells). This result can be explained by two possibilities: firstly, the overall pool of proliferating cells (BrdU^+^) is diminished, directly reducing the “raw material” available for neuronal differentiation. Alternatively, even when cells proliferate successfully, the inflammatory environment created by surgery may disfavor a neuronal fate, potentially steering newborn neurons toward gliogenesis or direct apoptosis (Monje et al. 2003; Zaben et al. 2021). Previous research suggests that ACh, acting via nicotinic and muscarinic receptors, directly influences NSCs and progenitor cells (Mohapel et al. 2005; Cooper‐Kuhn et al. 2004; Chen et al. 2024). To directly test whether cholinergic signaling could act on neural progenitor cells, we performed immunofluorescence staining and found that α7 nAChRs are expressed on DCX^+^ immature neurons and BrdU^+^ proliferating cells in the DG of aged mice (Figure S4). This provides a structural basis for a direct regulatory effect. Nevertheless, indirect effects through interneurons or glial cells cannot be excluded, and future cell‐type‐specific receptor knockout studies will be valuable.

The observed postoperative neurogenesis results reflect acute effects, and it remains unknown whether this suppression is transient or persistent. Longer observation timepoints are needed to determine if these cells are permanently lost. Retroviruses (e.g., GFP‐expressing RV) specifically infect and integrate into the genome of dividing cells. Like BrdU, they serve as a “birthdate marker,” but with a significant advantage: they enable long‐term, stable expression of a fluorescent protein (e.g., GFP) (Zhao et al. 2006). We found a reduction in newborn neurons at postoperative 4 weeks (decreased GFP^+^ cell number) alongside impaired neuronal development (reduced dendritic complexity). This suggests persistent microenvironmental abnormalities, insufficient neurotrophic support, or altered circuit activity (Jin et al. 2021; Paul et al. 2017; Song et al. 2012).

The MS/vDB represents a critical node vulnerable to age, anesthesia, surgical stress, and neuroinflammation (Xu et al. 2019; Chen et al. 2020). Our data confirm that anesthesia/surgery induces functional impairment in MS/vDB cholinergic neurons, likely contributing to reduced hippocampal ACh release. By combining chemogenetic activation of these neurons with local nAChR blockade, we demonstrated that the cognitive benefits of sustained MS/vDB activation are mediated through hippocampal nAChRs. This aligns with imaging studies in Alzheimer's disease showing basal forebrain atrophy and disruption of its hippocampal connections (Falangola et al. 2021; Liu et al. 2022). While our pharmacological experiments implicate nAChRs in the restorative effects, we acknowledge that muscarinic acetylcholine receptors (mAChRs) are also highly expressed in the hippocampus and may contribute. mAChRs, particularly M1 and M4 subtypes, are known to modulate hippocampal synaptic plasticity, neurogenesis, and memory processes (Li, Su, et al. 2022; Palacios‐Filardo et al. 2021). Given that MEC is a non‐selective nAChR antagonist, our findings do not exclude the possibility that mAChRs act in parallel or synergistically with nAChRs. Future studies employing selective mAChR antagonists or genetic deletion of specific receptor subtypes will be required to delineate the relative contributions.

We observed a clear dissociation between acute and chronic MS/vDB → DG manipulation: acute activation alleviated anxiety‐like behavior (increased center time in the OFT) but not memory deficits, whereas sustained stimulation significantly improved memory. Moreover, these therapeutic benefits of cholinergic activation are specifically amplified by the surgical insult. The differential effects of acute versus sustained activation may reflect receptor desensitization or circuit adaptation, with acute stimulation rapidly modulating affective circuits (e.g., amygdala) and prolonged activation required for structural plasticity and neurogenesis in the hippocampus. Notably, sustained MS/vDB → DG activation also promoted nAChR‐dependent newborn neuron survival, maturation, and hippocampal integration. However, the existence of functionally distinct MS/vDB cholinergic subpopulations (Wu et al. 2024)—for example, D28K‐positive neurons that co‐release ACh and GABA and project to ventral CA1 versus D28K‐negative neurons that release only ACh and target the dorsal DG (Li, Yu, et al. 2022)—calls for subtype‐specific dissection to enable precise therapeutic targeting.

Notably, the present findings that acute MS/vDB → DG cholinergic activation exerted anxiolytic‐like effects selectively in aged surgical mice, without altering basal anxiety in non‐surgical controls, appear to contradict the prevailing view that enhanced cholinergic signaling is anxiogenic in naive animals (Mineur et al. 2022). This discrepancy can be reconciled by circuit specificity, state‐dependent neuromodulation, and functional divergence among distinct septohippocampal subcircuits. Consistent with Mineur et al. (2022), we found that targeted chemogenetic activation of MS/vDB → DG projections did not alter anxiety‐like behaviors or memory performance in control mice, supporting that somatic or pathway‐specific cholinergic activation does not inherently evoke anxiogenic responses under physiological baseline conditions. Furthermore, McHugh et al. (2015) showed that selective immunotoxic depletion of hippocampal ACh (approximately 80% reduction in ChAT activity) did not affect baseline anxiety, indicating that tonic septohippocampal input is dispensable for basal anxiety.

The anxiogenic effects reported in prior studies mainly arose from broad or ventral hippocampal cholinergic manipulations, such as pan‐hippocampal terminal activation or retrograde DREADD recruitment of widespread hippocampal‐projecting cholinergic populations (Mineur et al. 2022). Emerging evidence has functionally dissociated these septohippocampal circuits: the MS/vDB → ventral hippocampus pathway drives anxiogenic responses, whereas the dorsal DG‐targeted projection primarily supports cognitive processing (Wu et al. 2024). Thus, our DG‐specific manipulation belongs to an intrinsically non‐anxiogenic subcircuit.

Critically, surgical insult in aged mice reduced hippocampal ACh release, establishing a cholinergic‐deficient pathological state. Acute MS/vDB → DG activation under this condition likely restores physiological cholinergic tone and normalizes DG circuit function, rather than inducing non‐physiological overactivation. This state‐dependent restorative pattern aligns with previous cholinergic studies. Mineur et al. (2022) reported that Gi‐DREADD‐mediated inhibition of MSDB cholinergic neurons exerted no behavioral effect at baseline but reversed physostigmine‐induced anxiety‐ and depression‐like behaviors. Similarly, McHugh et al. (2015) showed that chronic hippocampal ACh depletion did not alter basal anxiety, while transient behavioral deficits implied context‐dependent vulnerability to cholinergic perturbation. Furthermore, the MS/vDB → DG circuit is canonically implicated in contextual memory processing and pattern separation, rather than the direct regulation of innate anxiety (Knox and Keller 2016). Accordingly, the anxiolytic‐like phenotype observed in surgical mice may arise indirectly from improved hippocampal information processing and threat‐safe contextual discrimination.

In summary, the behavioral consequences of septohippocampal cholinergic signaling are governed by circuit topography, physiological versus pathological state, and temporal activation pattern. Our findings do not contradict existing literature but rather extend it: under hypocholinergic pathological conditions, even acute activation of the cognition‐oriented DG‐projecting cholinergic pathway can exert restorative behavioral benefits, including the attenuation of surgery‐elicited anxiety‐like behavior.

Several limitations should be acknowledged. First, our exploratory laparotomy model may not recapitulate other clinically relevant surgeries; more translational models will enhance clinical relevance. Second, while we established that MS/vDB → DG cholinergic signaling promotes neurogenesis, the precise molecular mechanisms—and how ACh differentially influences neural progenitors at various developmental stages—remain unclear. Third, OFT‐only anxiety assessment (single center time measure) may not capture multidimensional affective states. Future studies incorporating additional behavioral paradigms such as the elevated plus maze or light–dark box will be valuable to comprehensively evaluate the affective consequences of septohippocampal cholinergic modulation. Fourth, neurogenesis‐behavior correlation does not confirm causality; future studies employing specific interventions targeting NSCs and progenitors at defined stages will help clarify their role in cognitive recovery. Finally, absent contextual fear memory differences may reflect task sensitivity, circuit differences, testing order effects, or greater variability in aged mice; additional hippocampus‐dependent tasks (e.g., Morris water maze) will be necessary for full characterization.

In conclusion, our findings highlight the essential role of the MS/vDB → DG cholinergic circuit in mediating surgery‐induced memory decline and impaired neurogenesis in aged mice. Anesthesia and surgery suppress MS/vDB cholinergic neuron activity, reduce hippocampal ACh release, and disrupt neurogenesis—deficits that can be reversed by either local ACh potentiation or sustained pathway activation. These results position the MS/vDB‐DG cholinergic axis as a promising therapeutic target for preventing or treating POCD in the aging population, with potential translational implications for clinical practice.

## Author Contributions

Study conception and design: J.‐j.Y. and J.C. Conduct of experiments: L.L., Q.M., and X.H. Data analysis: J.Y. and N.D. Manuscript writing: L.L. Revisions of the manuscript: S.S., K.H., and J.‐j.Y. Approval of final version for publication: all authors.

## Funding

National Natural Science Foundation of China (U23A20421 to J.‐J.Y.) and the Scientific Research and Innovation Team of The First Affiliated Hospital of Zhengzhou University (ZYCXTD2023012 to J.‐J.Y.).

## Conflicts of Interest

The authors declare no conflicts of interest.

## Supporting information


**Figure S1:** Arterial blood gas analysis confirms comparable oxygenation between surgery and control groups.
**Figure S2:** Surgery induces cognitive deficits in aged mice.
**Figure S3:** Surgery impairs hippocampal long‐term potentiation in aged mice.
**Figure S4:** α7 nAChR is expressed on neural progenitor cells and immature neurons in the DG of aged mice.
**Figure S5:** Sustained chemogenetic activation of MS/vDB ChAT→DG cholinergic projections does not alleviate postoperative anxiety‐like behavior.

## Data Availability

Data are available in the main text and the Supporting Information S1, and further inquiries are available from the corresponding author on request.
