# Supplementary material for: Restoring Septohippocampal Cholinergic Signaling Rescues Surgery‐Induced Neurogenesis and Memory Deficits in Aged Mice
Source: Aging Cell. 2026 Jun 4;25(6):e70574. doi: 10.1111/acel.70574 (PMC13238679; doi:10.1111/acel.70574)
Supplement: Supplementary file 1 — Figure S1: Arterial blood gas analysis confirms comparable oxygenation between surgery and control groups. Figure S2: Surgery induces cognitive deficits in aged mice. Figure S3: Surgery impairs hippocampal long‐term potentiation in aged mice. Figure S4: α7 nAChR is expressed on neural progenitor cells and immature neurons in the DG of aged mice. Figure S5: Sustained chemogenetic activation of MS/vDB ChAT→DG cholinergic projections does not alleviate postoperative anxiety‐like behavior. [file ACEL-25-e70574-s001.docx]

**
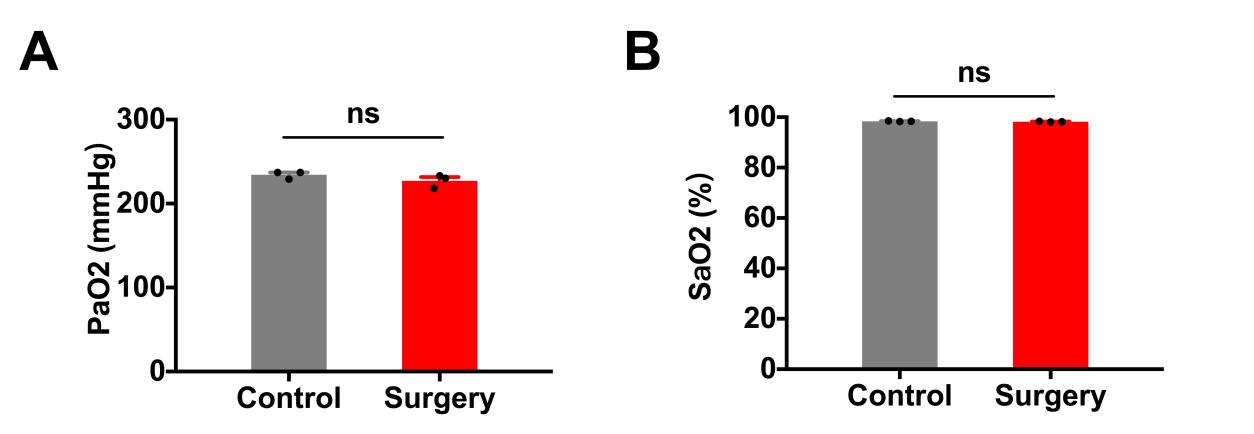
**

**Supplementary Figure 1 Arterial blood gas analysis confirms comparable oxygenation between surgery and control groups.**

**(A)** Partial pressure of arterial oxygen (PaO_2_) (Mann-Whitney test, *U* = 2, *P* = 0.300). **(B)** Arterial oxygen saturation (SaO_2_) (unpaired *t*-test, *t*(4) = 1.581, *P* = 0.189). *N* = 3 per group. Data are presented as mean ± SEM. ns, not significant.

**
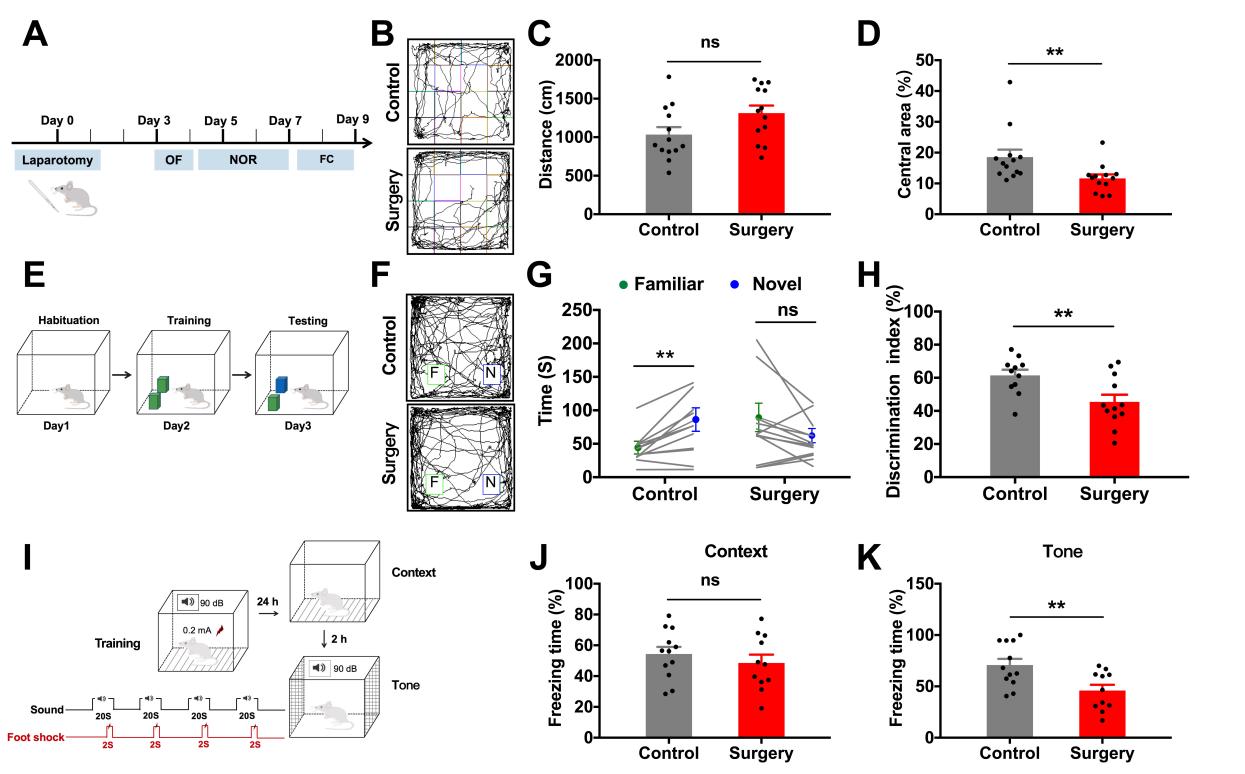
**

**Supplementary Figure 2 Surgery induces cognitive deficits in aged mice.**

**(A)** Schematic of the behavioral experimental timeline. **(B-D)** Open field test (OFT). **(B)** Representative movement traces. **(C)** Total distance (unpaired *t*-test, *t*(24) = 2.026, *P* = 0.054). **(D)** Time spent in the center zone (Mann-Whitney test, *U* = 25, *P* = 0.002). *N* = 13 per group. **(E)** Schematic of the novel object recognition (NOR) test. **(F)** Representative exploration traces. **(G)** Exploration time of novel versus familiar objects (control: Wilcoxon test, *W* = 58, *P* = 0.007; surgery: Wilcoxon test, *W* = -42, *P* = 0.110). **(H)** Discrimination index (DI) (unpaired *t*-test, *t*(21) = 2.861, *P* = 0.009). *N* = 11-12 per group. **(I)** Schematic of the fear conditioning (FC) test. **(J)** Contextual fear memory (unpaired *t*-test, *t*(21) = 0.821, *P* = 0.421). (K) Cued fear memory (unpaired *t*-test, *t*(21) = 2.964, *P* = 0.007). *N* = 11-12 per group. Data are presented as mean ± SEM. ***P* < 0.01.

**
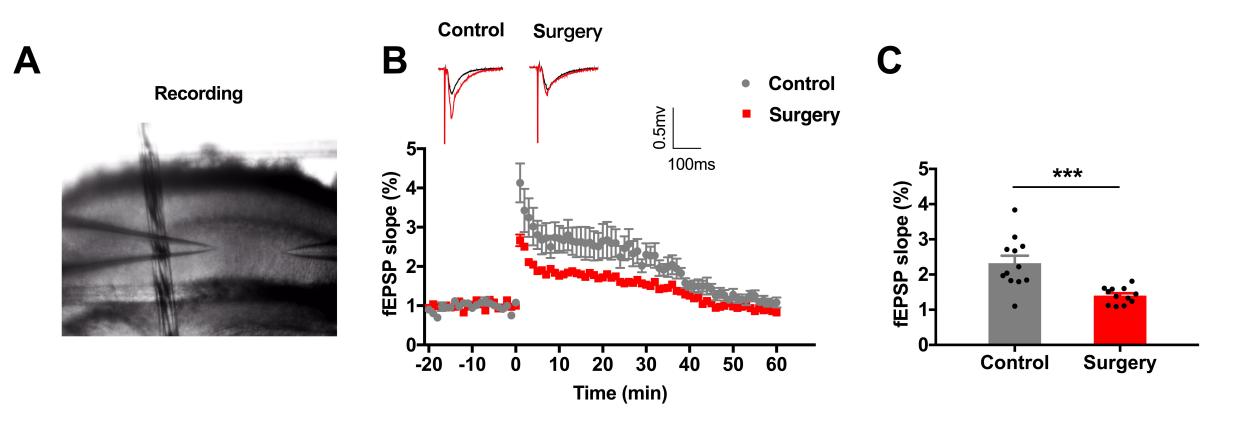
**

**Supplementary Figure 3 Surgery impairs hippocampal long-term potentiation in aged mice.**

1. Long-term potentiation (LTP) recording at hippocampal Schaffer collateral→CA1 synapses induced by 100 Hz tetanic stimulation. Representative fEPSP traces are shown. **(B, C)** Quantification of LTP levels (unpaired *t*-test, *t*(22) = 4.183, *P* = 0.0004). *N* = 12 slices from 4 mice per group. Data are presented as mean ± SEM. ****P* < 0.001.


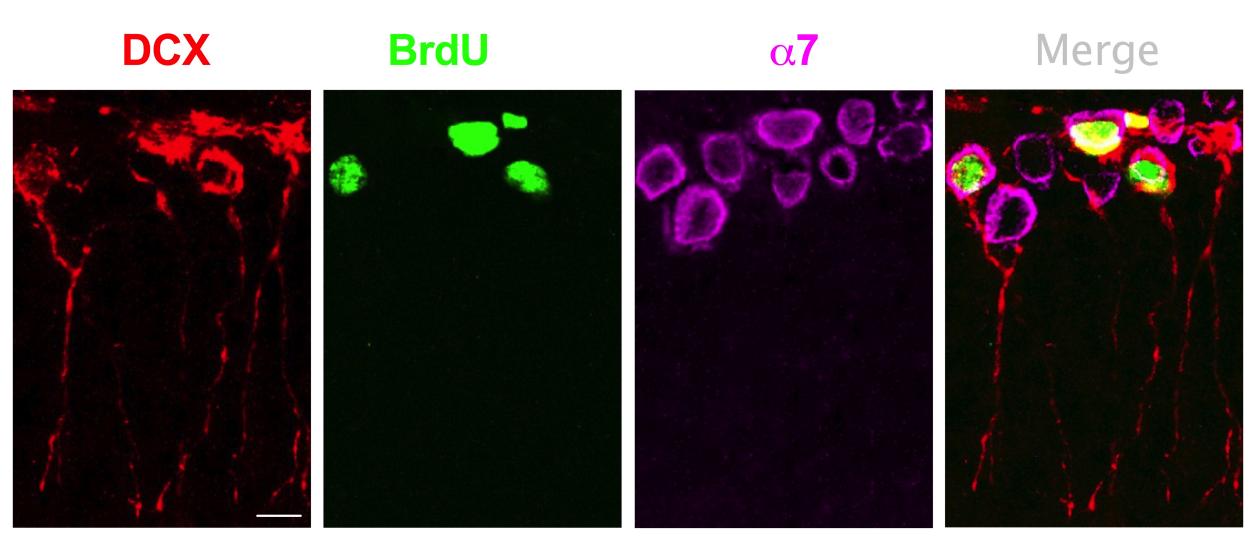


Supplementary Figure 4 α7 nAChR is expressed on neural progenitor cells and immature neurons in the DG of aged mice.

Representative triple immunofluorescence staining for DCX (red, immature neurons), BrdU (green, proliferating cells) and α7 nAChR (magenta). Scale bar: 20 μm.

**
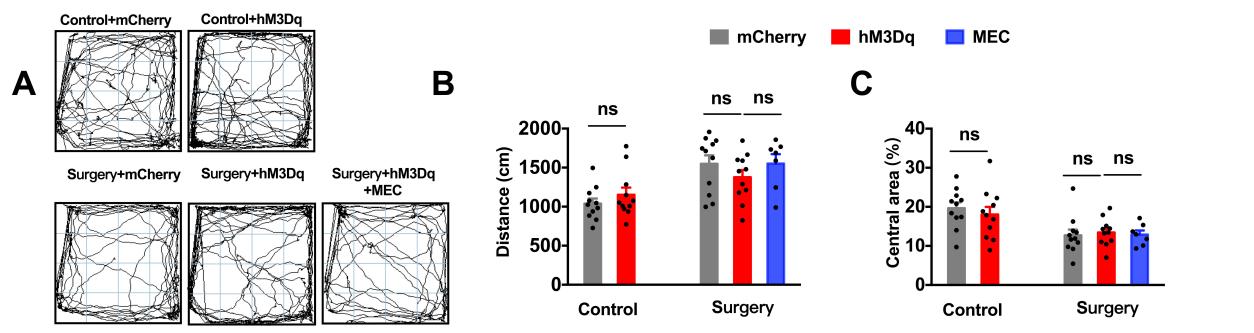
**

**Supplementary Figure 5 Sustained chemogenetic activation of MS/vDB ChAT**→**DG cholinergic projections does not alleviate postoperative anxiety-like behavior.**

1. Representative movement traces in the OFT. **(B)** Total distance traveled. Two-way ANOVA showed a significant main effect of surgery (*F*(1,40) = 16.56, *P* < 0.001), but no significant main effect of hM3Dq (*F*(1,40) = 0.108, *P* = 0.744) and no interaction (*F*(1,40) = 2.483, *P* = 0.123). Post-hoc analysis showed that hM3Dq did not significantly alter distance in surgery mice (*P* = 0.539) or in control mice (*P* = 0.054). One-way ANOVA among surgery groups showed no significant difference (*F*(2,21) = 0.948, *P* = 0.400). **(C)** Time spent in the center zone. Two-way ANOVA showed a significant main effect of surgery (*F*(1,40) = 14.26, *P* < 0.001), but no significant main effect of hM3Dq (*F*(1,40) = 0.078, *P* = 0.781) and no interaction (*F*(1,40) = 0.579, *P* = 0.451). One-way ANOVA among the three surgery groups showed no significant difference (*F*(2,21) = 0.100, *P* = 0.905). *N* = 7-11 per group. Data are presented as mean ± SEM. ns, not significant.
